# Ubiquitin E3 Ligase c-Cbl Is a Host Negative Regulator of Nef Protein of HIV-1

**DOI:** 10.3389/fmicb.2020.597972

**Published:** 2020-11-19

**Authors:** Hong-Guang Zhang, Jing Guo, Yukang Yuan, Yibo Zuo, Jin Liu, Li Zhu, Ying Miao, Xiangjie Chen, Lincong Jin, Fan Huang, Tengfei Ren, Jiuyi He, Weifeng Shi, Zhenke Wen, Chuanwu Zhu, Hui Zheng, Chunsheng Dong, Feng Qian

**Affiliations:** ^1^Institutes of Biology and Medical Sciences, Soochow University, Suzhou, China; ^2^Jiangsu Key Laboratory of Infection and Immunity, Soochow University, Suzhou, China; ^3^The Second Affiliated Hospital of Soochow University, The Affiliated Infectious Diseases Hospital of Soochow University, Suzhou, China; ^4^Department of Laboratory Medicine, The Third Affiliated Hospital of Soochow University, Changzhou, China

**Keywords:** c-Cbl, HIV-1, Nef, ubiquitination, E3 ubiquitin ligase

## Abstract

Nef is an accessory protein encoded by human immunodeficiency virus type-1 (HIV-1) and plays important roles in regulating HIV-1 infection and viral replication. Interestingly, HIV-1 Nef can promote degradation of numerous host proteins to disrupt cellular antiviral immune response. However, how HIV-1 Nef is degraded by host factors remains largely unexplored. Here, we identified c-Cbl as a host ubiquitin E3 ligase of HIV-1 Nef. We found that c-Cbl interacts with Nef and reduces protein levels of HIV-1 Nef. Further studies demonstrated that c-Cbl promoted Lys48-linked polyubiquitination of HIV-1 Nef, thus attenuating protein stability of HIV-1 Nef. Importantly, cellular c-Cbl ubiquitinated and degraded Nef proteins produced by HIV-1 NL4-3 virions, and ultimately attenuated HIV-1 virulence for infection of THP1 cells. This study reveals a ubiquitination and proteasome-dependent degradation mechanism of HIV-1 Nef protein, and could provide potential strategies for fighting against HIV-1.

## Introduction

In the long-term process of virus evolution, human immunodeficiency virus-1 (HIV-1) has developed many strategies to escape the immunosuppression of the host, which ultimately facilitates virus survival and subsequent replication (Strebel, 2013; Seissler et al., 2017; Yin et al., 2020). Among these strategies, HIV-1-encoded proteins widely hijack the signaling transduction activities of the host cells, and destroy the host immune defense to resist host antiviral responses. On the contrary, in the process of fighting against HIV-1, host cells have evolved various mechanisms to inhibit virus-mediated destruction effects on host immune activities (Zheng et al., 2012; Colomer-Lluch et al., 2018).

The HIV-1 genome encodes several structural proteins (Gag, Pol and Env) and regulatory proteins (Tat and Rev), as well as some accessory proteins (Nef, Vif, Vpr, and Vpu) (Saxena et al., 2019). Among the accessory proteins, Nef is a 27 kDa protein and known to play important roles in regulating host immune responses (Das and Jameel, 2005). The structure analysis indicates that Nef does not have an enzymatic activity, but it contains a flexible region which forms a large protein surface, thereby easily interacting with other proteins (Arold et al., 1997; Moroco et al., 2018). Recent human proteomics data have showed that at least 50 host proteins can interact with Nef (Jager et al., 2011). Interestingly, HIV-1 Nef has been demonstrated to regulate proteasomal degradation of host cell surface receptor CCR5 and CXCR4 (Sloan et al., 2010). In addition, HIV-1 Nef recruits AP-2, Alix, and β-COP to mediate degradation of the CD4 molecule (Schaefer et al., 2008; Stolp et al., 2009; Amorim et al., 2014; Ren et al., 2014; Pereira and daSilva, 2016). Similarly, HIV-1 Nef can downregulate SERINC5 via the endosome/lysosome system (Rosa et al., 2015; Kmiec et al., 2018; Shi et al., 2018; Jin et al., 2019). In addition, Nef induces HDAC6 degradation by an acidic/endosomal-lysosomal processing to counteract the antiviral activity of HDAC6 (Marrero-Hernández et al., 2019). Nef and E6AP can co-operate to promote p53 ubiquitination and degradation in order to suppress p53 dependent apoptosis (Ali et al., 2020). Although these findings uncover the roles of HIV-1 Nef in downregulating host proteins, how HIV-1 Nef protein is downregulated by host factors remains largely unexplored.

Cellular proteins can be degraded through the lysosomal and proteasomal pathways (Dunn, 1994). Ubiquitin-dependent proteasomal/lysosomal system plays crucial roles in regulating viral replication, protecting viruses from host immune system and countering host restriction factors (Klinger and Schubert, 2005; Li et al., 2016; Ren et al., 2016; Rojas and Park, 2019). Ubiquitin E3 ligases are central to recognize substrate proteins for ubiquitination modifications (Glickman and Ciechanover, 2002). Human genome contains more than 600 ubiquitin E3 ligases, many of which are responsible for ubiquitination of multiple substrate proteins (Glickman and Ciechanover, 2002; Li et al., 2008). Recent studies have reported that cellular ubiquitin E3 ligase Cyclin F interacts with HIV-1 Vif and restricts progeny virion infectivity by ubiquitination and proteasomal degradation of Vif (Augustine et al., 2017). Similarly, INSIG1 inhibits HIV-1 replication by degrading Gag (Zhang et al., 2019). In addition, HIV-1 gp120 protein can also be degraded through ubiquitination mechanisms (Bohl et al., 2013; Nityanandam and Serra-Moreno, 2014). However, the mechanism by which host ubiquitin E3 ligases ubiquitinate and degrade HIV-1 Nef protein remains elusive.

In the present study, we reported that an immunoregulatory ubiquitin E3 ligase c-Cbl binds to HIV-1 Nef protein. c-Cbl can enhance Nef K48-linked ubiquitination, thus promoting proteasome-dependent degradation of HIV-1 Nef. Importantly, host c-Cbl negatively regulates protein levels of Nef produced by HIV-1 (NL4-3) virions, which ultimately inhibits HIV-1 virulence for infection of human macrophages. This study promotes the understanding of host antiviral responses and could provide potential strategies for clinical HIV-1 therapy.

## Materials and Methods

### Cell Culture and Transfection

Human embryonic kidney 293T cells (HEK293T) were from ATCC. Cells were cultured in Dulbecco’s modified Eagle’s medium (DMEM; HyClone) at 37^°^C under 5% CO_2_. Culture medium was supplemented with 10% FBS, 100 μg/ml streptomycin and 100 U/ml penicillin. THP-1 cells (ATCC, TIB-202) were maintained in Roswell Park Memorial Institute 1640 medium (1640 RPMI; HyClone) supplemented with 10% fetal bovine serum (FBS), 2 mM L-glutamine, and 100 U/ml penicillin-streptomycin at 37°C in a humidified atmosphere with 5% CO_2_. Human PBMCs were from Dr. Zhenke Wen (Soochow University, China). Transient transfections were performed using LongTrans (UcallM, TF/07) according to the manufacturer’s instructions.

### Plasmids and Reagents

Plasmids expressing HIV-1 virus proteins, including FLAG-tagged Nef (FLAG-Nef), FLAG-tagged Vif (FLAG-Vif), FLAG-tagged gp120 (FLAG-gp120), and eGFP-tagged Gag (eGFP-Gag), were from Dr. Chuanwu Zhu (Fifth People’s Hospital of Soochow University, China). Myc-c-Cbl and Myc-Cbl-b were gifts from Dr. Jingping Zhang (Soochow University, China). Myc-c-Cbl C382A mutant was generated by Quick-Change site-Directed Mutagenesis Kit (Stratagene). HA-ubiquitin (HA-Ub), HA-R48K and HA-R63K (all lysins on the ubiquitin gene are mutated to arginines except the corresponding lysine) were described as previously (Guo et al., 2019; Yuan et al., 2020; Zuo et al., 2020). HIV-1 (pNL4-3), HIV-1 (ΔNef) and VSVG were from Dr. Chunsheng Dong (Soochow University, China). All shRNAs were constructed using the RNAi-Ready pSIREN-RetroQ-ZsGreen vector. The shc-Cbl targeting sequences were as follows:

shc-Cbl #1, 5′-gatccAGGAGAATTCTCAGCCTAGTTCAAG AGACTAGGCTGA GAATTCTCCTTTTTTTg-3′ and 5′-aatt cAAAAAAAGGAGAATTCTCAGCCTA GTCTCTTGAACTAG GCTGAGAATTCTCCTg-3′.

shc-Cbl #2, 5′-gatccACCAGATACCTACCAGCATTTCAAG AGAATGCTGGTA GGTATCTGGTTTTTTTg-3′ and 5′-aatt cAAAAAAACCAGATACCTACCAGC ATTCTCTTGAAATGC TGGTAGGTATCTGGTg-3′.

### RNA Isolation and RT-qPCR

Total RNAs were extracted from cells using the TRIzol reagent (Invitrogen). All cDNA was synthesized from 600 ng of total RNAs using the 5 × All-In-One RT Master Mix (Abcam, cat. no. G490). Then mRNA levels were analyzed by RT-qPCR using 2 × SYBR Green qRCR Master Mix (Selleck, cat. no. B21202). The primer sequences were listed as follows:

HIV-1 *nef*: forward, 5′-GCCTGGCTAGAAGCACAAGAA-3′ and reverse, 5′-GTGATGAAA TGCTAGGCGGC-3′.

Human *c-Cbl*: forward, 5′-CGCTAAAGAATAGCCCACCTT AT-3′ and reverse, 5′-ATGGCCT CCAGCCCAGAACTGAT-3′.

β*-actin*: forward, 5′-ACCAACTGGGACGACATGGAGA AA-3′ and reverse, 5′-ATAGCA CAGCCT GGATAGCA ACG-3′.

The relative expression of the target genes was normalized to β-actin mRNA and the results were analyzed from three independent experiments and were shown as the average mean ± s.d.

### HIV-1 Virus Stock Preparation and HIV-1 Infection

As to the replication competent HIV-1 viruses, HEK293T cells cultured on a 10 cm plate were transfected with 30 μg of proviral plasmids pHIV-1 (NL4-3) for production of HIV-1 virions. As to the Nef-deficient HIV-1 viruses (HIV-1-ΔNef), 25 μg of pHIV-luc ΔNef and 5 μg of pVSV-G were used for the production of VSV-G pseudotyped firefly luciferase reporter viruses. Cell culture medium was collected 48 h post-transfection, clarified at 3,500 rpm for 10 min, filtered through a 0.45-μm filter and concentrated by ultracentrifugation at 30,000 rpm for 1.5 h at 4^°^C. Aliquots were made and HIV-1 virions were stored at −80^°^C.

PMA (80 ng/ml)-activated THP-1 cells or PBMCs were infected with 0.5 or 1.0 m.o.i. of HIV-1 (NL4-3) viruses or HIV-luc (ΔNef) viruses for 6 h at 37^°^C in the presence of Polybrene (8 μg/ml) with intermittent mixing. Then cells were washed twice by 1XPBS. PBMCs were cultured in complete medium supplemented with recombinant human IL-2 (Research and development, R&D) at 20 units/ml until harvested. Culture supernatants from the infected cells were collected to determine virus production by the p24 antigen capture ELISA.

### Cycloheximide Pulse Chase Assay

FLAG-Nef protein stability was determined by cycloheximide (CHX) pulse chase assay. Briefly, HEK293T cells were co-transfected with two vectors to express FLAG-Nef and Myc-c-Cbl. Twenty-four hours after transfection, cells were treated with DMSO or CHX (100 μg/ml) for different times. Then cells were harvested and subjected to analysis by western blots. The densities of immunoblotting bands were analyzed with the Image J program, and the dates were analyzed from three independent experiments and were shown as the average mean ± s.d.

### Immunoprecipitation and Immunoblotting

All cells harvested in Non-idet P-40 lysis buffer (pH 7.4) containing 50 mM Tris-HCL, 150 mM NaCl, 1% Non-idet P-40, 1 mM EDTA, 50 μg/ml PMSF. The denatured proteins were subjected to SDS-PAGE and transferred to polyvinylidene difluoride (PVDF) membranes (Millipore), followed by blocking with 5% skim milk for 1 h at room temperature. Then the membrane was incubated with the specific primary antibodies, including anti-FLAG (F7425; Sigma), anti-HA (ab9110; Abcam), anti-ubiquitin (sc-8017; Proteintech), anti-Myc (M20002H; Abmart), anti-GAPDH (AB-M-M001; Hangzhou Goodhere Biotechnology), anti-α-Tubulin (66031-1-Ig; Proteintech), anti-eGFP (sc-8334; Santa Cruz), anti-HIV-1-Nef (ab42358; Abcam) and anti-c-Cbl (D4E10; Cell Signaling Technology). After that, the PVDF membrane was washed three times with PBST (1 × PBS with 1% Tween 20), followed by incubation with the HRP-conjugated goat anti-mouse or goat anti-rabbit (Bioworld) secondary antibodies for 1 h at room temperature. The membrane was finally visualized with the ECL Prime (Thermo Fisher Scientific).

When protein ubiquitination was examined, RIPA lysis buffer (Beyotime) was used to harvest cells and N-ethylmaleimide (10 mM) was added into the lysis buffer. After immunoprecipitation, the immunoprecipitates were washed by high-salt (500 mM NaCl) washing buffer for three times and subsequently by normal washing buffer (150 mM NaCl) for twice. Then the immunoprecipitates were analyzed by western blot.

### Mass Spectrometry (MS)

HEK293T cells were transfected with empty vectors or a vector to express FLAG-Nef. Forty-eight hours after transfection, cells were harvested in 1 mL Non-idet P-40 lysis buffer. FLAG M2 affinity gel (A2220; Sigma-Aldrich) was used to pull down FLAG-Nef and its interacting proteins from whole cell lysates. SDS-PAGE gels were stained with Coomassie Brilliant Blue. The gel bands from control and experimental samples were carefully excised, followed by digestion using trypsin. The resulting tryptic peptides were purified by C18 Zip Tip and then were analyzed using an Orbitrap Elite hybrid mass spectrometer (Thermo Fisher Scientific) coupled with a Dionex LC. Spectral data were analyzed by the Proteome Discoverer 1.4 against a UniProt protein database.

### Immunofluorescence Microscopy

HEK293T cells were transfected with two vectors to express Myc-c-Cbl and FLAG-Nef. After washing by 1 × PBS, cells were fixed in 4% paraformaldehyde on ice and then permeabilized with 0.1% Triton X-100 and blocked with 3% BSA. Next, cells were incubated with an anti-Myc antibody and an anti-FLAG antibody in 1% BSA. After washing three times with 1 × PBS, cells were stained with 488 goat anti-mouse IgG (Alexa Fluor, A11001) or 594 goat anti-Rabbit IgG (Alexa Fluor, A11012). Cells nuclei were stained with DAPI. The fluorescent images were captured with the Nikon A1 confocal microscope.

### Statistical Analysis

Group comparisons were analyzed by a two-tailed Student *t-*test for statistical significance analysis. Values of ^∗^*P* < 0.05, ^∗∗^*P* < 0.01, and ^∗∗∗^*P* < 0.001 were considered statistically significant. NS, not significant. Data were analyzed using the Graph Pad Prism 7.0.

### Data Availability

The raw data supporting the conclusions of this article will be made available by the authors, without undue reservation.

## Results

### HIV-1 Nef Interacts With a Host Ubiquitin E3 Ligase c-Cbl

**FIGURE 1:**
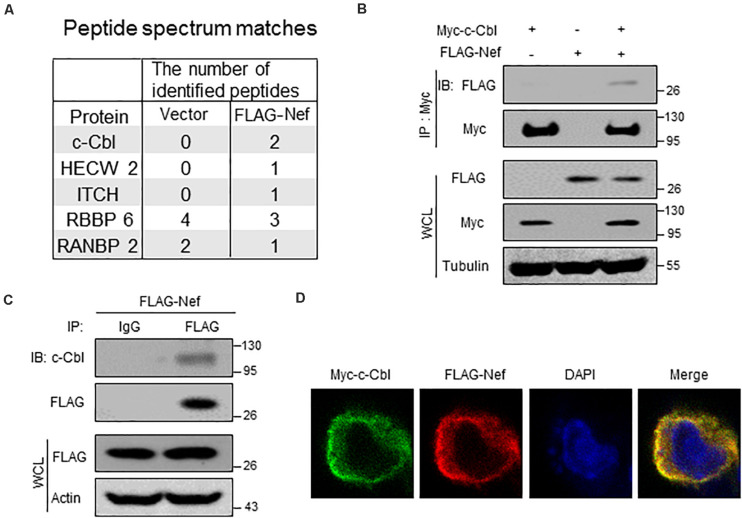
HIV-1 Nef interacts with a host ubiquitin E3 ligase c-Cbl. **(A)** HEK293T cells were transfected with empty vectors (Vector) or a vector to express FLAG-Nef. Immunoprecipitation was performed using the FLAG affinity gel. The immunoprecipitates were subjected to mass spectrum analysis and the potential ubiquitin E3 ligases interacting with FLAG-Nef were shown. **(B)** Immunoprecipitation analysis of the interaction between c-Cbl and Nef in HEK293T cells co-transfected with Myc-c-Cbl and FLAG-Nef plasmids. WCL: whole cell lysates. **(C)** HEK293T cells were transfected with a vector to express FLAG-Nef. Immunoprecipitation was performed using the FLAG affinity gel, and then endogenous c-Cbl was detected by immunoblotting with an anti-c-Cbl antibody. **(D)** Immunofluorescence analysis of the co-localization of Myc-c-Cbl and FLAG-Nef in HEK293T cells. Data are representative of three independent experiments with similar results **(B,C)**.

To study how HIV-1 Nef proteins are degraded in host cells, we first performed mass spectrum analysis to identify the potential Nef-interacting proteins. Our data showed that several cellular ubiquitin E3 ligases, including c-Cbl, HECW2, ITCH, could interact with Nef protein (Figure 1A). Thus, we next carried out immunoprecipitation analysis to confirm their interaction. We noticed that among the three potential ubiquitin E3 ligases, c-Cbl could be able to interact with Nef, as shown by the interaction between Myc-c-Cbl and FLAG-Nef in cells (Figure 1B). Furthermore, we observed that endogenous c-Cbl can also interact with FLAG-Nef (Figure 1C). Moreover, by immunofluorescence microscopy we observed that Myc-c-Cbl and FLAG-Nef have obvious co-localization in cells (Figure 1D). Together, these findings suggested that HIV-1 Nef protein could interact with cellular ubiquitin E3 ligase c-Cbl. Further analysis of the interaction between endogenous c-Cbl and Nef protein produced by HIV-1 virions was shown in the section of Figure 5.

### c-Cbl Is a Cellular Negative Regulator of HIV-1 Nef Protein

**FIGURE 2:**
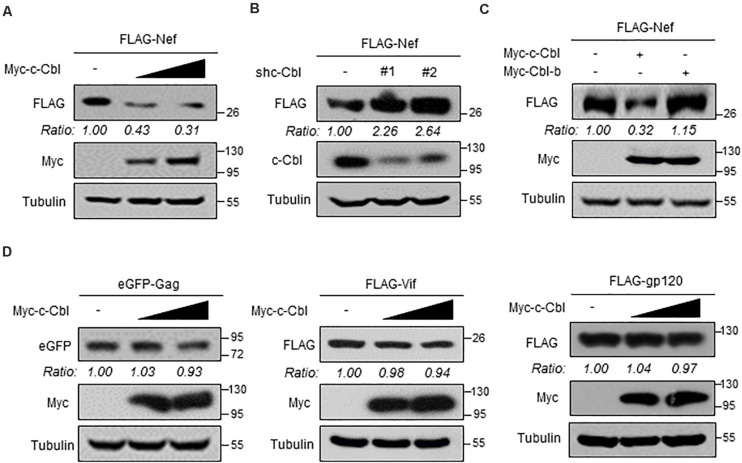
c-Cbl is a cellular negative regulator of HIV-1 Nef protein. **(A)** HEK293T cells were co-transfected with the vectors to express FLAG-Nef and increased amounts (0.5 and 1.0 μg) of Myc-c-Cbl. Whole cell extracts were subjected to immunoblotting as indicated. **(B)** HEK293T cells were co-transfected with the vectors to express FLAG-Nef and control shRNAs (−) or two shRNAs against c-Cbl (shc-Cbl, #1 and #2). Seventy-two hours after transfection, whole cell extracts were analyzed by immunoblotting using indicated antibodies. **(C)** HEK293T cells were co-transfected with the vectors to express FLAG-Nef and either Myc-c-Cbl or Myc-Cbl-b. Whole cells extracts were analyzed by immunoblotting using indicated antibodies. **(D)** HEK293T cells were transfected with either eGFP-Gag, or FLAG-Vif, or FLAG-gp120 plasmids, together with empty vectors (−) or increased amounts of Myc-c-Cbl plasmids. Whole cell extracts were analyzed by immunoblotting using indicated antibodies. Data are representative of three independent experiments with similar results **(A–D)**.

Given that the ubiquitin E3 ligase c-Cbl could interact with Nef, we wondered whether c-Cbl is capable of regulating Nef protein levels. To address this question, HEK293T cells were transfected with two vectors to express FLAG-Nef and Myc-c-Cbl. Our data showed that overexpression of c-Cbl remarkably downregulated FLAG-Nef protein levels in a dose-dependent manner (Figure 2A). Next, we used two specific shRNAs against human c-Cbl (shc-Cbl) to knock down endogenous c-Cbl. The results showed that knockdown of c-Cbl dramatically promoted FLAG-Nef protein expression in cells (Figure 2B). Furthermore, we analyzed the effect of Cbl-b on FLAG-Nef protein levels. Cbl-b is highly conserved in the N-terminal region with c-Cbl. Interestingly, Cbl-b-mediated ubiquitination usually does not result in protein degradation. Here, we found that Cbl-b did not affect the levels of FLAG-Nef (Figure 2C).

It has been reported that several proteins encoded by HIV-1, including Gag, Vif and gp120, undergo ubiquitination-dependent degradation in host cells. Thus, we determined whether c-Cbl could affect protein levels of these HIV-1 encoded proteins. To this end, HEK293T cells were transfected with either eGFP-Gag, or FLAG-Vif, or FLAG-gp120 plasmids, together with Myc-c-Cbl plasmids. Our data showed that c-Cbl did not significantly affect the levels of these HIV-1 encoded proteins (Figure 2D). In summary, these findings demonstrated that cellular c-Cbl is a negative regulator of HIV Nef protein expression.

### c-Cbl Lowers HIV-1 Nef Protein Stability

**FIGURE 3:**
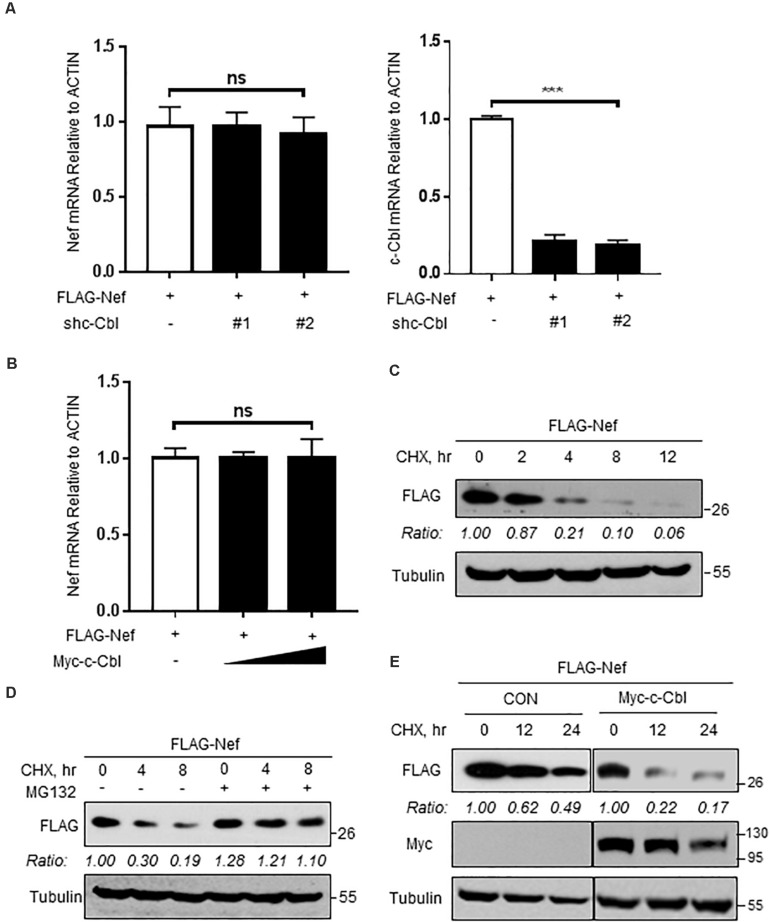
c-Cbl lowers HIV-1 Nef protein stability. **(A)** The relative mRNA levels of Nef and c-Cbl were measured by RT-qPCR in HEK293T cells co-transfected with the vectors to express FLAG-Nef and control shRNAs (−) or shc-Cbl (#1 and #2). **(B)** The relative mRNA levels of Nef were measured by RT-qPCR in HEK293T cells co-transfected with the vectors to express FLAG-Nef and empty vectors (−) or Myc-c-Cbl. **(C)** HEK293T cells were transfected with a vector to express FLAG-Nef. Twenty-four hours after transfection, cells were treated with CHX (100 μg/ml) for 0, 2, 4, 8, and 12 h. The protein levels of Nef were analyzed by immunoblotting. **(D)** HEK293T cells transfected with FLAG-Nef plasmids were pretreated with DMSO or MG132 (10 μM) for 2 h and then were treated with CHX (100 μg/ml) for 0, 4, and 8 h. The protein levels of Nef were analyzed by immunoblotting. **(E)** HEK293T cells were transfected with either empty vectors (CON) or Myc-c-Cbl plasmids, together with FLAG-Nef plasmids. Then cells were treated with CHX for 0, 12, and 24 h. Whole cell extracts were analyzed by immunoblotting using the indicated antibodies (left). Data were analyzed by GraphPad Prism 7 (right). NS, not significant (*P* > 0.05) and ***P < 0.001 (two-tailed unpaired Student’s *t*-test). Data are representative of three independent experiments **(C–E)**, or shown as mean and s.d. of three biological replicates **(A,B)**.

Based on the above findings, we next wanted to know whether c-Cbl downregulates Nef protein levels by regulating Nef transcriptional expression. To this end, HEK293T cells were co-transfected with the vectors to express FLAG-Nef and shc-Cbl. The results showed that knockdown of c-Cbl by two specific shc-Cbl did not affect Nef mRNA level (Figure 3A). In addition, c-Cbl overexpression did not affect the levels of Nef mRNA (Figure 3B). These data suggested that c-Cbl-mediated downregulation of Nef was not due to the inhibition of Nef transcriptional expression. Thus, we next utilized cycloheximide (CHX), which is an inhibitor of protein biosynthesis, to determine HIV-1 Nef protein stability in cells by CHX pulse chase assay (Figure 3C). We found that a proteasome inhibitor MG132 blocked FLAG-Nef degradation under conditions of CHX treatment (Figure 3D), suggesting that Nef proteins undergo proteasome-dependent degradation. Furthermore, we observed that overexpression of c-Cbl significantly lowered Nef protein stability (Figure 3E). These findings suggested that c-Cbl downregulates HIV-1 Nef protein levels by affecting Nef protein stability.

### c-Cbl Promotes K48-Linked Ubiquitination and Proteasome-Dependent Degradation of HIV-1 Nef Protein

**FIGURE 4:**
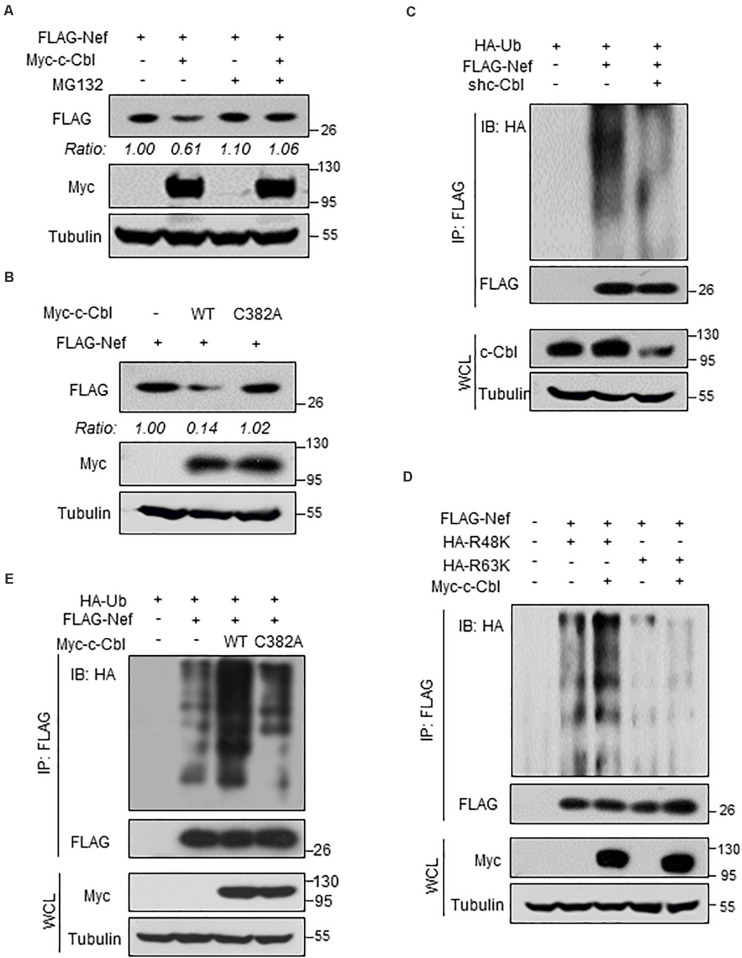
c-Cbl promotes K48-linked ubiquitination and proteasome- dependent degradation of HIV-1 Nef protein. **(A)** HEK293T cells transfected with FLAG-Nef and (or) Myc-c-Cbl plasmids were treated with or without MG132 (10 μM) for 12 h before harvest. The protein levels of Nef were analyzed by immunoblotting as indicated. **(B)** HEK293T cells were transfected with the vectors to express FLAG-Nef and either Myc-c-Cbl wild type (WT) or Myc-c-Cbl C382A mutant. Nef protein levels were analyzed by immunoblotting as indicated. **(C)** HEK293T cells were transfected with control shRNAs (−) or shc-Cbl (#1) plasmids, together with FLAG-Nef and HA-Ub plasmids as indicated. FLAG-Nef proteins were immunoprecipitated by FLAG beads, and FLAG-Nef ubiquitination levels were analyzed by immunoblotting. **(D)** HEK293T cells were transfected with FLAG-Nef and/or Myc-c-Cbl plasmids, together with HA-Ub-K48 only (R48K) or HA-Ub-K63 only (R63K) plasmids. FLAG-Nef proteins were immunoprecipitated by FLAG beads, and then FLAG-Nef ubiquitination levels were analyzed by immunoblotting. **(E)** HEK293T cells were transfected with FLAG-Nef and Myc-c-Cbl (WT or C382A mutant) plasmids, together with HA-Ub plasmids. FLAG-Nef proteins were immunoprecipitated by FLAG beads and then FLAG-Nef ubiquitination levels were analyzed by immunoblotting. Data are representative of three independent experiments with similar results **(A–E)**.

Given that c-Cbl lowers HIV-1 Nef protein stability, we further analyzed the underlying mechanism. MG132 is a proteasome inhibitor, which is widely used to analyze proteasome-dependent degradation of cellular proteins. We found that MG132 treatment blocked c-Cbl-mediated downregulation of HIV-1 Nef protein (Figure 4A), suggesting that c-Cbl downregulates Nef protein levels via the proteasome pathway. Furthermore, we constructed the c-Cbl C382A mutant, which has been demonstrated to be an ubiquitin E3 ligase inactive mutant of c-Cbl. Our results showed that the c-Cbl C382A mutant lost the ability to downregulate Nef proteins (Figure 4B), suggesting that c-Cbl-mediated degradation of Nef proteins is dependent on the E3 ligase activity of c-Cbl.

Given that c-Cbl regulates proteasome-dependent degradation of Nef proteins, we next determined whether c-Cbl regulates Nef protein ubiquitination. This result showed that knockdown of c-Cbl markedly inhibited polyubiquitination of FLAG-Nef protein (Figure 4C). Two types of ubiquitination modifications, Lysine 48 (K48)-linked and Lysine 63 (K63)-linked ubiquitination, have been extensively studied and mostly used for analysis of protein ubiquitination. We found that c-Cbl overexpression significantly promoted K48-linked but not K63-linked polyubiquitination of HIV-1 Nef protein (Figure 4D). Importantly, our results showed that the ubiquitin E3 ligase inactive mutant of c-Cbl, c-Cbl-C382A, was not able to promote ubiquitination of FLAG-Nef proteins (Figure 4E), suggesting that c-Cbl-mediated ubiquitination of Nef proteins is dependent on the ubiquitin E3 ligase activity of c-Cbl. It is well-known that K48-linked polyubiquitination of proteins usually promotes protein degradation. Thus, these findings suggest that cellular c-Cbl, as a ubiquitin E3 ligase, promotes K48-linked polyubiquitination of HIV-1 Nef protein, which subsequently results in Nef protein degradation through the proteasome pathway.

### c-Cbl Expression Is Upregulated During HIV-1 Infection

**FIGURE 5:**
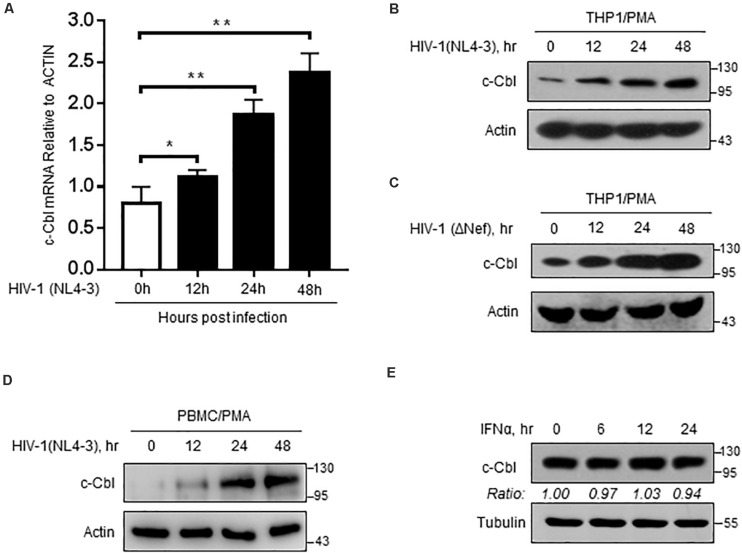
c-Cbl expression is upregulated during HIV-1 infection. **(A)** RT-qPCR analysis of c-Cbl mRNA levels in PMA-activated THP-1 cells infected with HIV-1 (NL4-3) virions for 0, 12, 24, and 48 h. **(B,C)** Immunoblotting analysis of c-Cbl protein levels in PMA-activated THP-1 cells infected with HIV-1 (NL4-3) virions **(B)** or HIV-1 (ΔNef) virions **(C)** for 0, 12, 24, and 48 h. **(D)** PBMCs from uninfected donors were activated with PMA (80 ng/ml), and then infected with 0.5 moi of HIV-1 (NL4-3) virions for 0, 12, 24, and 48 h. Whole cell extracts were subjected to immunoblotting as indicated. **(E)** Immunoblotting analysis of c-Cbl protein levels in HEK293T cells treated with IFNα (1,000 IU/ml) for 0, 6, 12, and 24 h. *P < 0.05, **P < 0.01 (two-tailed unpaired Student’s t-test). Data are shown as mean and s.d. of three biological replicates **(A)**, or representative of three independent experiments *(B–E)*.

We further utilized an infectious HIV-1 clone, HIV-1 (NL4-3), to produce HIV-1 virions in cells. Interestingly, we noticed that c-Cbl transcriptional expression was upregulated during HIV-1 infection (Figure 5A). In line with the observation, c-Cbl protein levels increased in PMA-treated THP1 cells infected with HIV-1 (Figure 5B). Similarly, we noticed that Nef-deficient HIV-1 virions can still promote expression of c-Cbl, suggesting that this effect is not dependent on Nef protein (Figure 5C). Importantly, c-Cbl protein in PBMCs (human peripheral blood mononuclear cells) from uninfected donors was upregulated by HIV-1 viral virions (Figure 5D). However, type-I interferons did not promote c-Cbl expression (Figure 5E). Taken together, these findings suggested that during HIV-1 infection host cells can enhance c-Cbl expression to fight against HIV-1.

### c-Cbl Regulates HIV-1-Encoded Nef Protein Levels and HIV-1 Virions

**FIGURE 6:**
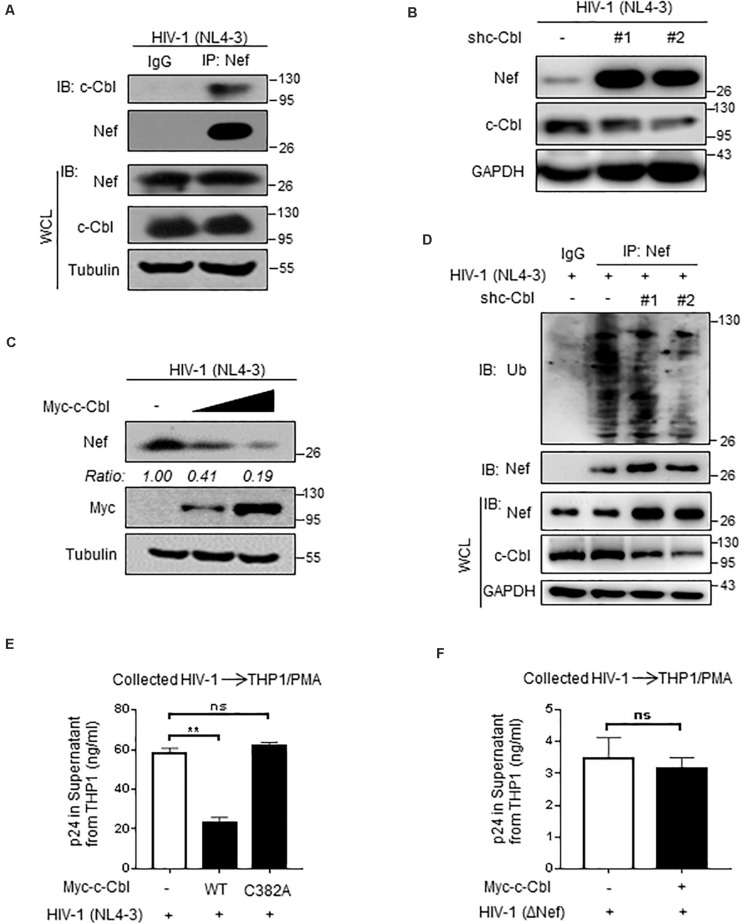
c-Cbl regulates HIV-1-encoded Nef protein levels and HIV-1 virions. **(A)** HEK293T cells were transfected with an infectious HIV-1 (NL4-3) constructs for 48 h to produce HIV-1 virions. Immunoprecipitation was performed to analyze the interaction between endogenous c-Cbl and HIV-1 (NL4-3)-produced Nef in cells. **(B)** HEK293T cells were transfected with HIV-1 (NL4-3) constructs and either control shRNAs (−) or two shc-Cbl (#1 and #2) plasmids. Whole cell extracts were subjected to immunoblotting as indicated. **(C)** HEK293T cells were transfected with HIV-1 (NL4-3) constructs and Myc-c-Cbl plasmids to produce HIV-1 virions. The protein levels of Nef produced by HIV-1 (NL4-3) virions were analyzed by immunoblotting as indicated. **(D)** HEK293T cells were transfected as **(B)**. Immunoprecipitation and immunoblotting were performed to analyze ubiquitination levels of HIV-1 Nef by an anti-Ub antibody. **(E)** HEK293T cells were transfected with an infectious HIV-1 (NL4-3) constructs, together with empty vectors (−) or Myc-c-Cbl (WT) or Myc-c-Cbl C382A plasmids, to produce HIV-1 virions. Forty-eight hours after transfection, HIV-1 virions were collected from the culture supernatant and then the same amount of HIV-1 virus was used to infect THP1 cells activated by PMA. The culture supernatant was collected to analyze HIV-1 p24 levels by p24 antigen capture ELISA. **(F)** HEK293T cells were transfected with HIV-1-luc (ΔNef) and VSV-G constructs, together with empty vectors (−) or Myc-c-Cbl plasmids, to produce HIV-1 ΔNef virions. Forty-eight hours after transfection, HIV-1 (ΔNef) virions were collected from the culture supernatant and were used to infect PMA-activated THP1 cells. The HIV-1 p24 levels were determined as **(E)**. NS, not significant (*P* > 0.05), **P < 0.01 (two-tailed unpaired Student’s *t*-test). Data are representative of three independent experiments **(A–D)**, or shown as mean and s.d. of three biological replicates **(E,F)**.

We further found that HIV-1-produced Nef protein can interact with cellular c-Cbl (Figure 6A), which is consistent with the aforementioned results in Figure 1. Importantly, HIV-1 (NL4-3) can produce higher levels of Nef proteins in c-Cbl-knockdown cells (Figure 6B). Consistently, overexpression of c-Cbl significantly inhibited expression levels of Nef protein produced by HIV-1 (NL4-3) (Figure 6C). Mechanistically, we found that knockdown of c-Cbl by two specific shRNAs inhibited cellular ubiquitination levels of Nef protein produced by HIV-1 (NL4-3) (Figure 6D), demonstrating that cellular c-Cbl promotes ubiquitination of HIV-1-produced Nef protein and in turn results in downregulation of Nef protein.

Given that Nef deficiency can result in attenuated virulence of HIV-1 for infection of macrophages or T cells, we employed human THP1 cells pretreated with PMA to observe HIV-1 virulence. We found that HIV-1 virions from wild type c-Cbl-overexpressing cells had much lower virulence for infection of THP1 cells, as shown by decreased levels of p24 protein, a commonly used marker to assess the levels of HIV-1 virions, in culture supernatant of THP1 cells (Figure 6E). However, the ubiquitin E3 ligase inactive C382A mutant of HIV-1 lost the ability to lower the virulence of HIV-1 virions (Figure 6E), suggesting that c-Cbl regulates HIV-1 virulence in an ubiquitin E3 ligase activity-dependent manner. Furthermore, we observed that overexpression of c-Cbl cannot affect the virulence of Nef-deficient HIV-1 virions (Figure 6F), which indicates that c-Cbl attenuates HIV-1 virulence by regulating Nef. Taken together, these findings demonstrated that cellular c-Cbl negatively regulates HIV-1 Nef protein levels and virulence of HIV-1 virions.

## Discussion

HIV-1 Nef has been demonstrated to be involved in downregulating a number of host proteins to antagonize immune responses. However, how host immune factors inhibit HIV-1 Nef remains elusive. This study revealed that c-Cbl, which has been reported to be involved in host immune activities, can target HIV-1 Nef protein and downregulate the levels of HIV-1 Nef protein in cells. Further studies demonstrated the detailed mechanism: c-Cbl promotes K48-linked ubiquitination and proteasomal degradation of HIV-1 Nef proteins dependently on its ubiquitin E3 ligase activity. Importantly, host c-Cbl can downregulate Nef proteins produced by HIV-1 NL4-3 virions, which results in attenuated virulence of HIV-1 for infection of human macrophages. Thus, this study revealed an important antiviral mechanism by which the host ubiquitin E3 ligase c-Cbl restricts HIV-1 Nef protein and HIV-1 virulence.

It has been documented that ubiquitin-proteasome or lysosomal system participates in regulating HIV-1 life cycle (Klinger and Schubert, 2005). HIV-1-encoded proteins have been reported to undergo ubiquitination and degradation by one or multiple host E3 ligase(s). For example, MDM2 can downregulate HIV-1 Vif levels by inducing its degradation in a proteasome-dependent manner (Izumi et al., 2009). In addition, Cyclin F also downregulates HIV-1 Vif by ubiquitination and proteasomal degradation (Augustine et al., 2017). BCA2/Rabring7 can target HIV-1 Gag for lysosome-dependent degradation of HIV-1 Gag protein (Nityanandam and Serra-Moreno, 2014). Similarly, INSIG1 can also degrade HIV-1 Gag (Zhang et al., 2019). Here, we found that c-Cbl induces ubiquitination and proteasome-dependent degradation of HIV-1 Nef. It will be interesting to explore other ubiquitination-mediated regulation of HIV-1 Nef in the future.

c-Cbl is initially recognized as a proto-oncogene with widespread mutations in hematopoietic malignancies (Naramura et al., 2011). Recent study has revealed that c-Cbl plays important roles in antiviral innate immunity. c-Cbl inhibited innate immunity by promoting K48-linked ubiquitination at Lys813 and proteasome-dependent degradation of RIG-I protein (Chen et al., 2013). c-Cbl could inhibit production of type-I interferon (IFN-I) by enhancing K48-linked ubiquitination and degradation of IRF3 protein (Ritterhoff et al., 2016). These reports suggested that c-Cbl is a negative regulator of host immune activities. Here, we found that c-Cbl can downregulate HIV-1 Nef proteins and therefore inhibit HIV-1 virulence. Thus, this study revealed a positive role of c-Cbl in regulating antiviral immunity against the specific HIV-1 virus.

As discussed above, c-Cbl plays complex roles in innate antiviral immunity, which could either promote or inhibit viral infection. Interestingly, we further found that during HIV-1 infection c-Cbl expression was upregulated, suggesting a positive feedback of host cells for fighting against HIV-1. However, IFN-I cannot stimulate c-Cbl expression, which implies that other signaling pathways activated by viruses, such as NF-κB or subsequent secreted cytokines, could contribute to the increase in c-Cbl levels. In fact, given that HIV-1 employs numerous strategies to disable IFNs signaling, it could be inefficient for host cells to utilize IFNs signaling to stimulate c-Cbl expression. In addition, it can also be observed that viruses stimulate expression of some cellular proteins independently of IFN-I signaling. For example, viral infection stimulated HOIP protein expression dependently on the NF-κB but not IFN-I pathways (Zuo et al., 2020). Cellular OTUD1 proteins were upregulated by viral infection in a NF-κB-dependent manner (Zhang et al., 2018). Thus, it will be interesting to study the detailed mechanisms of c-Cbl upregulation by HIV-1 infection in the future. In addition, our data showed that Nef-deficient HIV-1 can still stimulate c-Cbl expression, suggesting HIV-1-mediated c-Cbl upregulation is independent of Nef. It is reasonable that HIV-1 Nef protein will not induce host c-Cbl to downregulate itself. Together, these findings revealed a host general mechanism for fighting HIV-1 infection.

Recent studies have clearly demonstrated that Nef can downregulate host SERINC5, which ultimately promotes HIV-1 infection (Rosa et al., 2015; Shi et al., 2018; Featherstone and Aiken, 2020; Staudt and Smithgall, 2020). SERINC5 is an intrinsic membrane protein that restricts HIV-1 infectivity when incorporated into HIV-1 budding virions, while HIV-1 Nef prevents SERINC5 incorporation into the virions. Given that we demonstrated the role of c-Cbl in regulating HIV-1 Nef protein levels, we believe that c-Cbl-mediated downregulation of HIV-1 Nef could inhibit HIV-1 infection through facilitating SERINC5-mediated antiviral activity. Consistent with this idea, we observed that c-Cbl did inhibit infectivity of wild type but not Nef-deficient HIV-1 virions. Certainly, other regulatory factors could be also involved in c-Cbl-mediated inhibition of HIV-1 infectivity. Taken together, these findings promote understanding of the roles of both Nef and c-Cbl in regulating HIV-1 infectivity.

In this study, we noticed that another member of the Cbl family, Cbl-b, cannot downregulate HIV-1 Nef protein. Both c-Cbl and Cbl-b share highly conserved regions in their N-terminal halves, whereas their C-terminal regions are less well conserved (Thien and Langdon, 2005). It has been reported that they could have different consequences for their substrates. Unlike c-Cbl, Cbl-b-mediated ubiquitination does not often induce degradation of its substrate proteins. In addition, some studies have reported opposing effects between c-Cbl and Cbl-b (Thien and Langdon, 2005). For example, c-Cbl overexpression inhibited, while Cbl-b enhanced, TCR-induced activation of NFAT (Zhang et al., 1999). Here, we reported that c-Cbl but not Cbl-b inhibited HIV-1 Nef protein and HIV-1 virulence.

Interestingly, a recent study reported that during infection with murine AIDS virus, c-Cbl protein expression was dramatically reduced in the in T- and B-lymph node cells of infected mice, while it remained normal in the thymus (Trebak et al., 1998), suggesting that HIV could antagonize c-Cbl-mediated negative regulation on HIV-1-encoded proteins at certain stage of infection. Further detailed studies are required for the understanding of the interaction between host c-Cbl and HIV-1. In summary, our study revealed host c-Cbl-mediated inhibition of HIV-1 and could help to understand the host-pathogen interaction.

## Data Availability Statement

The proteomic data was uploaded onto the ProteomeXchange repository (the number is: MassIVE MSV000086376). The information can directly be downloaded from: ftp://massive.ucsd.edu/MSV000086376/. The title is: Identification of the potential Nef-interacting proteins (10.25345/C5G49N) [dataset license: CC0 1.0 Universal (CC0 1.0)]. The Dataset identifier: PXD022208.

## Author Contributions

H-GZ and JG performed all experiments and edited manuscript. YY, YZ, JL, YM, XC, LJ, FH, TR, and JH provided technical support. LZ, ZW, and WS contributed materials and important discussion. CZ, HZ, CD, and FQ designed the experiments and made substantial contributions to the study. All authors have given final approval of the version to be published and agree to be accountable for all aspects of the work.

## Conflict of Interest

The authors declare that the research was conducted in the absence of any commercial or financial relationships that could be construed as a potential conflict of interest.
